# Heterotrimeric G-protein Signaling Is Critical to Pathogenic Processes in *Entamoeba histolytica*

**DOI:** 10.1371/journal.ppat.1003040

**Published:** 2012-11-15

**Authors:** Dustin E. Bosch, Adam J. Kimple, Robin E. Muller, Patrick M. Giguère, Mischa Machius, Francis S. Willard, Brenda R. S. Temple, David P. Siderovski

**Affiliations:** 1 Department of Pharmacology, The University of North Carolina at Chapel Hill, Chapel Hill, North Carolina, United States of America; 2 Department of Biochemistry & Biophysics, The University of North Carolina at Chapel Hill, Chapel Hill, North Carolina, United States of America; 3 R. L. Juliano Structural Bioinformatics Core, The University of North Carolina at Chapel Hill, Chapel Hill, North Carolina, United States of America; 4 Department of Physiology & Pharmacology, West Virginia University School of Medicine, Robert C. Byrd Health Sciences Center, Morgantown, West Virginia, United States of America; University of Virginia Health System, United States of America

## Abstract

Heterotrimeric G-protein signaling pathways are vital components of physiology, and many are amenable to pharmacologic manipulation. Here, we identify functional heterotrimeric G-protein subunits in *Entamoeba histolytica*, the causative agent of amoebic colitis. The *E. histolytica* Gα subunit EhGα1 exhibits conventional nucleotide cycling properties and is seen to interact with EhGβγ dimers and a candidate effector, EhRGS-RhoGEF, in typical, nucleotide-state-selective fashions. In contrast, a crystal structure of EhGα1 highlights unique features and classification outside of conventional mammalian Gα subfamilies. *E. histolytica* trophozoites overexpressing wildtype EhGα1 in an inducible manner exhibit an enhanced ability to kill host cells that may be wholly or partially due to enhanced host cell attachment. EhGα1-overexpressing trophozoites also display enhanced transmigration across a Matrigel barrier, an effect that may result from altered baseline migration. Inducible expression of a dominant negative EhGα1 variant engenders the converse phenotypes. Transcriptomic studies reveal that modulation of pathogenesis-related trophozoite behaviors by perturbed heterotrimeric G-protein expression includes transcriptional regulation of virulence factors and altered trafficking of cysteine proteases. Collectively, our studies suggest that *E. histolytica* possesses a divergent heterotrimeric G-protein signaling axis that modulates key aspects of cellular processes related to the pathogenesis of this infectious organism.

## Author Summary

*Entamoeba histolytica* causes an estimated 50 million intestinal infections and 100,000 deaths per year worldwide. Here, we identify functional heterotrimeric G-protein subunits in *Entamoeba histolytica*, constituting a signaling pathway which, when perturbed, is seen to regulate multiple cellular processes required for pathogenesis. Like mammalian counterparts, EhGα1 forms a heterotrimer with EhGβγ that is dependent on guanine nucleotide exchange and hydrolysis. Despite engaging a classical G-protein effector, EhRGS-RhoGEF, EhGα1 diverges from mammalian Gα subunits and cannot be classified within mammalian Gα subfamilies, as highlighted by distinct structural features in our crystal structure of EhGα1 in the inactive conformation. To identify roles of G-protein signaling in pathogenesis-related cellular processes, we engineered trophozoites for inducible expression of EhGα1 or a dominant negative mutant, finding that G-protein signaling perturbation affects host cell attachment and the related process of contact-dependent killing, as well as trophozoite migration and Matrigel transmigration. A transcriptomic comparison of our engineered strains revealed differential expression of known virulence-associated genes, including amoebapores and cytotoxic cysteine proteases. The expression data suggested, and biochemical experiments confirmed, that cysteine protease secretion is altered upon G-protein overexpression, identifying a mechanism by which pathogenesis-related trophozoite behaviors are perturbed. In summary, *E. histolytica* encodes a vital heterotrimeric G-protein signaling pathway that is likely amenable to pharmacologic manipulation.

## Introduction

GTP-binding proteins (G-proteins) are important transducers of cellular signaling [1]. Heterotrimeric G-proteins are composed of three distinct subunits (Gα, Gβ, and Gγ) and typically coupled to seven-transmembrane domain, G-protein coupled receptors (GPCRs). Gα binds guanine nucleotide while Gβ and Gγ form an obligate heterodimer [1]. Conventionally, Gα forms a high-affinity binding site for Gβγ when Gα is in its inactive GDP-bound state. Activated receptor acts as a guanine nucleotide exchange factor (GEF) for Gα, releasing GDP and allowing subsequent GTP binding. The binding of GTP causes a conformational change in three flexible “switch” regions within Gα, resulting in Gβγ dissociation. Gα·GTP and freed Gβγ independently activate downstream effectors, such as adenylyl cyclases, phospholipase C isoforms, and Rho-family guanine nucleotide exchange factors (RhoGEFs) to modulate levels of intracellular second messengers [1], [2]. ‘Regulator of G-protein signaling’ (RGS) proteins generally serve as inhibitors of Gα-mediated signaling [3]; however, one class of RGS protein, the RGS-RhoGEFs, serve as positive “effectors” for activated Gα signal transduction [2], [4].

Heterotrimeric G-protein signaling has provided a wealth of targets amenable to pharmacologic manipulation, most prevalent being the GPCR itself [5]. Heterotrimeric G-proteins in mammals regulate processes as diverse as vision, neurotransmission, and vascular contractility [1], [5]. Heterotrimeric G-proteins in non-mammalian organisms also exhibit a wide range of functions; for example, pheromone and nutrient sensing in yeast [6], hydrophobic surface recognition in the rice blast fungus [7], and cellular proliferation and chemical gradient sensing in the slime mold *Dictyostelium discoideum* [8], [9].

*Entamoeba histolytica* causes an estimated 50 million infections and 100,000 deaths per year worldwide [10]. *E. histolytica* infection is endemic in countries with poor barriers between drinking water and sewage; however, outbreaks also occur among travelers and susceptible subpopulations in developed countries [11]. Upon cyst ingestion, the amoeba may colonize the human colon. Although the majority of infections are asymptomatic (*e.g.* ref [12]), a fraction results in symptomatic amoebic colitis. Migratory *E. histolytica* trophozoites attach to intestinal epithelial cells through a Gal/Gal-NAc lectin [13]. Amoebae subsequently kill host cells through a number of mechanisms, including secretion of cell-perforating amoebapores [14], [15] and release of cytotoxic cysteine proteases [16].

*E. histolytica* has been studied for more than 50 years, and some of the signaling pathways important for pathogenesis have been identified. Several transmembrane kinases have been implicated cellular proliferation, phagocytosis, and the establishment of intestinal infection [17], [18], [19]. Calcium signaling is also involved in phagocytosis; for instance, calcium binding protein 1 (EhCaBP1) modulates the actin cytoskeleton at phagocytic cups and, together with the EhC2PK kinase, is involved in phagosome maturation [20], [21], [22], [23]. Rho family GTPases and their activating exchange factors are also involved in a variety of pathogenic processes, including migration, phagocytosis, and surface receptor capping [24], [25], [26], [27]. The related Rab family small GTPases control trafficking and maturation of cellular vesicles, and are implicated in processes such as phagocytosis and cysteine protease secretion [28], [29], [30], [31].

However, many *E. histolytica* signaling components, and thus potential targets for therapeutic intervention, remain under-studied. For example, recent sequencing of the *E. histolytica* genome identified multiple potential cell signal transduction components; *e.g.*, 307 putative protein kinases representing all seven eukaryotic kinase families have been identified, including receptor tyrosine kinases [17], [32]. In this paper, we describe genetic, structural, and biochemical data establishing the identity of *E. histolytica* heterotrimeric G-protein signal transduction components as well as their regulatory roles in pathogenesis-related behaviors of *E. histolytica*.

## Results

### Identification of *E. histolytica* heterotrimeric G-protein subunits

By a BLAST sequence similarity search with human Gα_i1_ (E-value cutoff of 10^−30^), we identified a single gene in *E. histolytica* encoding a putative Gα subunit (EhGα1; AmoebaDB EHI_140350) also present in the related *E. dispar*, *E. invadens*, *E. moshkovskii*, and *E. terrapinae*. One Gβ subunit was also identified (AmoebaDB EHI_000240) by sequence similarity to human Gβ1 (E-value cutoff of 10^−30^), termed EhGβ1 (Fig. S1B). As Gβ subunits form obligate heterodimers with short Gγ polypeptides or Gγ-like (GGL) domains [3], we also searched for putative Gγ-encoding genes. Based on sequence similarity with *S. cerevisiae* Ste18 and *D. discoideum* gpgA, together with alignment of candidate protein sequences and identification of key functional residues, we identified two putative Gγ-encoding genes named EhGγ1 and EhGγ2; these two open-reading frames (in the NCBI *E. histolytica* genomic contigs AAFB02000029.1 and AAFB02000157.1, respectively) each possess a C-terminal CAAX-box that specifies isoprenylation in conventional Gγ subunits [33].

To determine whether these G-protein subunits are expressed in *E. histolytica*, we amplified trophozoite mRNA using quantitative RT-PCR. Transcripts of *EhGα1*, *EhGβ1*, and *EhGγ1* were all detected, along with the housekeeping gene glyceraldehyde-3-phosphate dehydrogenase (GAPDH; AmoebaDB EHI_167320) (Fig. S2).

### Functional assessments of *E. histolytica* G-protein subunits

**Figure 1:**
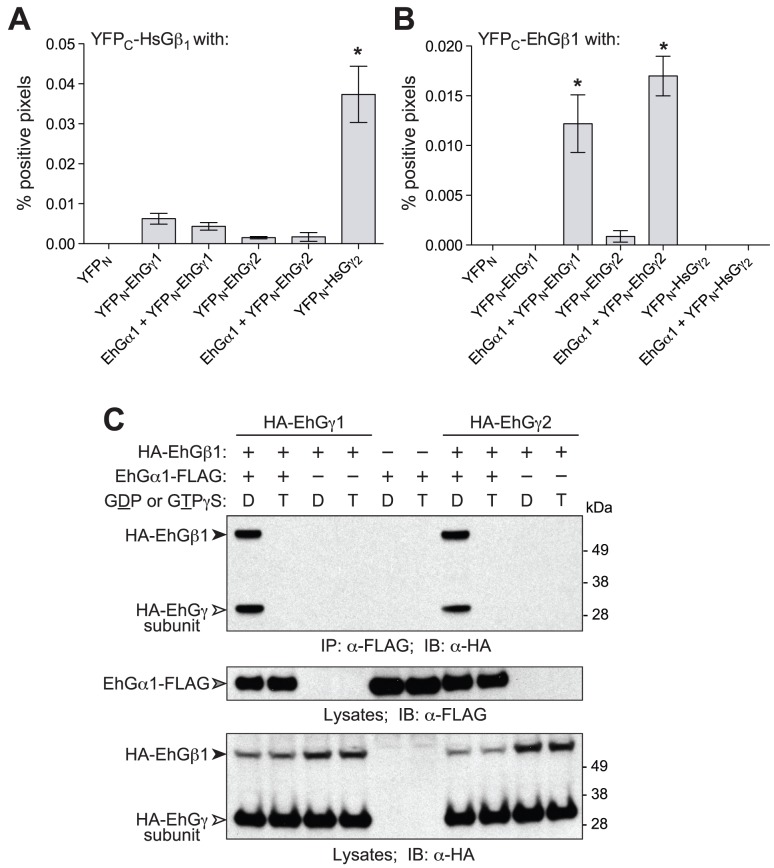
*E. histolytica* G-protein subunits form a heterotrimer in a nucleotide-dependent manner. Interactions between Gβ and Gγ subunits were detected with split-YFP protein complementation in COS-7 cells. (**A**) Human Gβ1 heterodimerized with human Gβ2, but not with *E. histolytica* Gγ subunits. (**B**) EhGβ1 interacts with EhGγ1 or EhGγ2 when co-expressed with EhGα1. (**C**) G-protein heterotrimer formation in the presence of excess GDP (“D”) or the non-hydrolyzable GTP analog, GTPγS (“T”), was examined with co-immunoprecipitation. EhGβ1 and EhGγ1 or EhGγ2 interacted selectively with EhGα1 in its GDP-bound, inactive state. Error bars represent standard error of the mean for three experiments. * represents statistically significant difference from zero, as determined by 95% confidence intervals excluding zero.

To determine whether the identified EhGα1, EhGβ1, EhGγ1, and EhGγ2 subunits form conventional heterodimeric (Gβγ) and heterotrimeric (Gα·GDP/Gβγ) complexes, bimolecular fluorescence complementation and co-immunoprecipitation assays were performed (Fig. 1A, B). The N-terminal half of yellow fluorescent protein (YFP_N_) was fused to EhGγ1 and EhGγ2 open reading frames while the C-terminus of YFP (YFP_C_) was fused to EhGβ1. Only when YFP_C_ and YFP_N_ are fused to interacting proteins will the fluorescent protein fold and function correctly [34], as shown with the human G-protein subunits Gβ_1_ and Gγ_2_ (Fig. 1A). Significant cellular fluorescence was observed only when EhGα1 was co-transfected with YFP_C_-EhGβ1 and either YFP_N_-EhGγ1 or YFP_N_-EhGγ2 (Fig. 1B). Example epifluorescence micrographs are show in Figure S3. As expected, co-expression of YFP_C_ alone with any of the YFP_N_-fusions did not yield measurable cellular fluorescence. EhGβ1/γ1 and EhGβ1/γ2 dimers were found to interact with EhGα1 only in the presence of GDP (and not GTPγS) (Fig. 1C), consistent with canonical Gα·GDP/Gβγ interaction selectivity.

**Figure 2:**
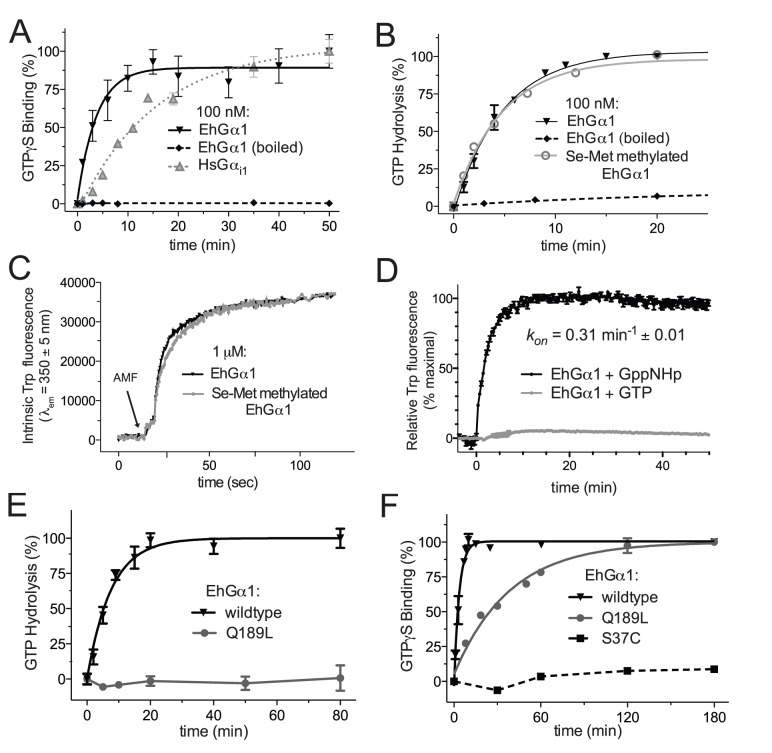
EhGα1 cycles between an active, GTP-bound state and an inactive, GDP-bound state. (**A**) EhGα1 bound non-hydrolyzable GTPγS as determined by radionucleotide binding. The observed exchange rate, *k_obs_* = 0.27 min^−1^ ±0.06, indicates faster spontaneous GDP release than human Gα_i1_ (*k_obs_* = 0.06 min^−1^ ±0.01). (**B**) EhGα1 hydrolyzed GTP[γ-^32^P] at 0.21 min^−1^ ±0.02, as determined by single turnover hydrolysis assays. No difference was observed for selenomethionine, lysine-methylated EhGα1 used for crystallization. (**C**) EhGα1 changes conformation upon binding the transition state mimetic aluminum tetrafluoride. Intrinsic EhGα1 fluorescence following excitation at 285 nm increases upon activation, reflecting burial of a conserved tryptophan on switch 2 (Trp-196). (**D**) EhGα1 adopts an active switch conformation upon addition of the non-hydrolyzable GTP analog GppNHp, as reflected by increased intrinsic tryptophan fluorescence. The kinetics of GppNHp-mediated activation are consistent with the kinetics of radiolabeled GTP analog binding (Fig. 1A). In contrast, addition of hydrolyzable GTP does not result in EhGα1 activation, indicating that nucleotide exchange, rather than GTP hydrolysis, is the rate-limiting step in the nucleotide cycle of EhGα1. (**E, F**) Two EhGα1 point mutants were profiled for effects on nucleotide cycle. The dominant negative S37C possessed negligible GTP binding. The constitutively active Q189L bound but did not hydrolyze GTP. Error bars in all panels represent standard error of the mean.

To determine if *E. histolytica* Gα binds and hydrolyzes GTP, EhGα1 was purified from *E. coli*. Spontaneous nucleotide exchange (as measured by [^35^S]GTPγS binding) was determined to be 0.27 min^−1^ and 0.064 min^−1^ at 30°C for EhGα1 and human Gα_i1_ respectively (Fig. 2A). The observed EhGα1 exchange rate is comparable to that of Gα_o_ [35], one of the faster spontaneous exchangers among mammalian Gα subunits. EhGα1 exhibited an intrinsic GTP hydrolysis rate of 0.21 min^−1^ at 20°C (Fig. 2B), comparable to rates previously observed for human Gα_i1_ and Gα_i3_ under the same conditions (*e.g.*, ref. [36]).

Trp-196 in switch 2 of EhGα1 is universally conserved among Gα subunits (*e.g.*, Fig. S1A) and translocates to a hydrophobic pocket upon Gα activation – an event which is easily measured as a dramatic change of intrinsic tryptophan fluorescence in select Gα subunits that lack multiple additional tryptophan residues (*e.g.*, ref. [37]). Exposure to the activating reagent AlF_4_^−^ and magnesium (AMF) increases tryptophan fluorescence (Fig. 2C), and thus EhGα1 appears to assume a similar, activated switch conformation as conventional Gα subunits. Since the measured rates of EhGα1 nucleotide exchange (0.27 min^−1^ at 30°C) and hydrolysis (0.21 min^−1^ at 20°C) were on the same order of magnitude, we tested whether hydrolysis was rate-limiting, as seen for the *A. thaliana* Gα protein, AtGPA1 [38]. While EhGα1 assumes an activated conformation upon exposure to the non-hydrolyzable GTP analog, GppNHp, as indicated by intrinsic tryptophan fluorescence, addition of hydrolyzable GTP was insufficient to activate EhGα1 (Fig. 2D). Thus, nucleotide exchange is the rate-limiting step in the steady-state nucleotide cycling of EhGα1, as for mammalian Gα subunits, indicating that activation likely relies on GEF-stimulated exchange.

### EhGα1 functional mutants

To further characterize EhGα1 activation properties and provide tools for probing G protein function in *E. histolytica* trophozoites, we mutated presumed key residues of the nucleotide-cycling function of EhGα1. Gln-189 in switch 2 (Fig. S1A) is predicted to coordinate the critical nucleophilic water responsible for γ-phosphoryl group hydrolysis [39]. Mutation of this residue to leucine in mammalian Gα subunits results in inability to hydrolyze GTP even in the presence of GTPase-accelerating proteins [35]. The corresponding EhGα1(Q189L) mutation abolished the ability of EhGα1 to hydrolyze GTP (Fig. 2E), suggesting a conserved role for the switch 2 Gln-189 residue in orienting the nucleophilic water. The Q189L mutant also exhibited a slower rate of 0.026 min^−1^ (95% C.I., 0.021–0.031 min^−1^) for GTPγS binding compared to wildtype (Fig. 2F), likely due to the slow rate of GTP dissociation in the absence of hydrolysis. Co-immunoprecipitation experiments demonstrated that EhGα1(Q189L) did not interact with EhGβ1/γ2 dimers when cell lysates were incubated with either GDP or GTPγS (Fig. S4), consistent with a state of constitutive activation.

In a mutagenesis screen [40], the mammalian Gα residue corresponding to Ser-37 of EhGα1, when mutated to cysteine, was identified as constitutively binding Gβγ irrespective of whether presented with GDP or GTP analogs. We hypothesized that we could create an EhGα1 variant that constitutively binds GDP by mutating Ser-37 to cysteine. The EhGα1(S37C) mutant showed no appreciable GTPγS binding (Fig. 2F), consistent with dominant negative behavior due to disrupted GTP/Mg^2+^ binding. Given that the EhGα1(S37C) mutant did not bind GTP, single turnover assays were not possible with this mutant. However, EhGα1(S37C) was observed to form a heterotrimer with EhGβ1/γ2 in the presence of either GDP or GTPγS (Fig. S4), consistent with dominant negative character.

### Evolutionary analysis of EhGα1 and identification of a putative effector

**Figure 3:**
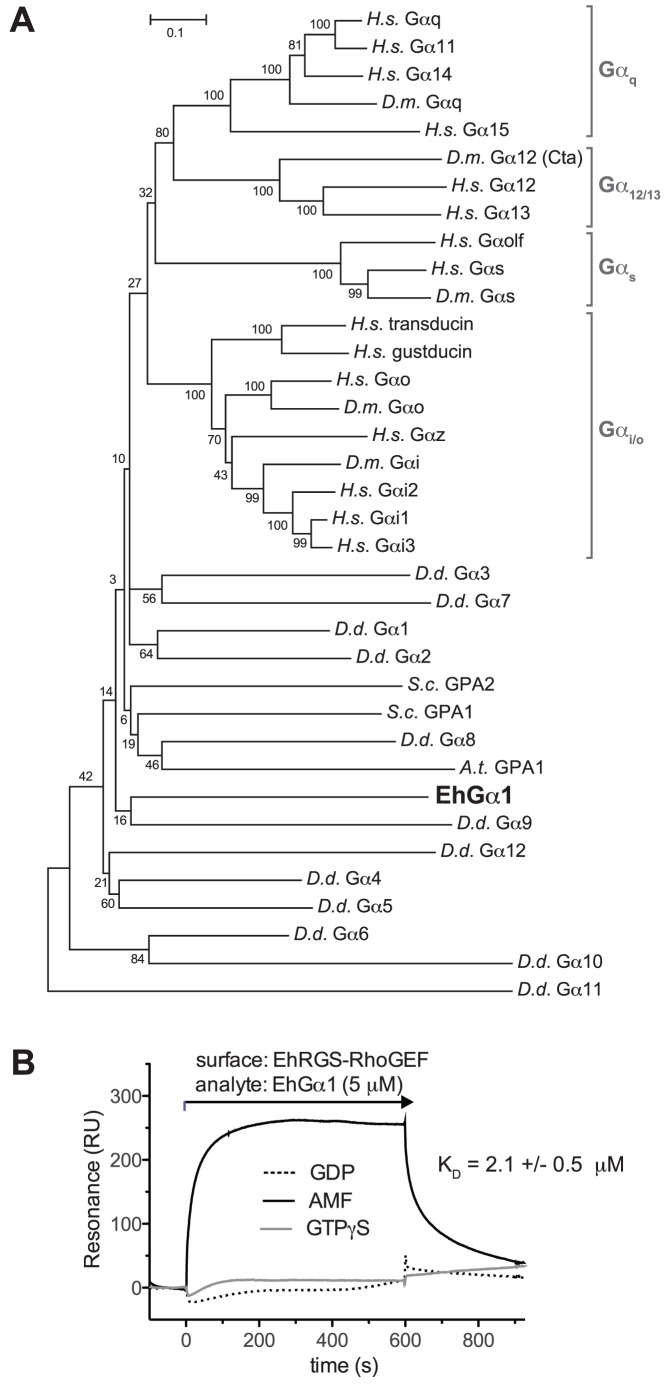
Evolutionary relationship of Gα subunits and identification of EhRGS-RhoGEF as a putative effector for activated EhGα1. (**A**) Gα subunit protein sequences from *E. histolytica*, *D. discoideum* (D.d.), *A. thaliana* (A.t.), *S. cerevisiae* (S.c.), *D. melanogaster* (D.m), and *H. sapiens* (H.s.) were aligned and a bootstrapping consensus phylogram created using MEGA5 [41]. Bootstrap values are indicated at each branch point. EhGα1 is distantly related to metazoan Gα subunits, specifically the adenylyl cyclase stimulatory Gα_s_, adenylyl cyclase inhibitory Gα_i/o_, phospholipase Cβ coupled Gα_q_, and RGS-RhoGEF activating Gα_12/13_ subfamilies. (**B**) Recombinant EhRGS-RhoGEF protein was immobilized on a surface plasmon resonance chip and EhGα1 protein flowed over in one of two nucleotide states. The EhRGS-RhoGEF biosensor bound EhGα1 selectively in the activated, GDP·AlF_4_^−^-bound state (AMF).

In an attempt to identify the Gα subunit family that EhGα1 most closely resembles, we generated a phylogenetic tree comparing Gα subunits from multiple species (Fig. 3A) using MEGA5 [41]. EhGα1 is only distantly related to the metazoan Gα subunits, including the Gα_12/13_ subfamily that couples to RGS-RhoGEFs. EhGα1 is most similar to *D. discoideum* Gα9, a Gα subunit involved in cellular proliferation [8], although low bootstrap values in the phylogram region surrounding EhGα1 indicate uncertain topology. EhGα1 also has similarity to *A. thaliana* GPA1 and the yeast Gα subunits, GPA1 and GPA2, the latter with roles in pheromone response and nutrient sensing, respectively [6]. The *A. thaliana* GPA1 regulates diverse processes, such as transpiration and cellular proliferation in response to glucose [42], [43]. We also calculated sequence similarity between EhGα1 and an array of human Gα subunits based upon multiple sequence alignments. In calibrating this method, the five known Gα subunits of *Drosophila melanogaster* showed sequence similarity patterns allowing facile classification into each of the Gα subfamilies (Gα_s_, Gα_i/o_, Gα_q_, Gα_12/13_) (Fig. S5A); however, both EhGα1 and GPA1 from *Saccharomyces cerevisiae* exhibited low sequence similarities to each of the human Gα subfamilies (Fig. S5B). EhGα1 exhibits the lowest similarity to each mammalian Gα tested, implying a likely early evolutionary departure from an ancestral Gα.

The *E. histolytica* genome was found to encode an RGS domain-containing RhoGEF (AmoebaDB EHI_010670; named EhRGS-RhoGEF) with distant homology to the RGS-RhoGEF effectors of mammalian Gα_12/13_ subunits; no other canonical Gα effector proteins, such as adenylyl cyclases or phospholipase Cβ isoforms, were identified. The transcript encoding EhRGS-RhoGEF was detected within trophozoite mRNA using quantitative RT-PCR (Fig. S2). Recombinant EhRGS-RhoGEF was therefore expressed and purified from *E. coli*; as measured by surface plasmon resonance, immobilized EhRGS-RhoGEF protein was found to bind EhGα1 selectively in its GDP·AlF_4_^−^ (AMF) nucleotide state (Fig. 3B). This selective binding is consistent with a putative EhGα1 effector function for EhRGS-RhoGEF, yet occurs in the absence of significant homology of EhGα1 to the mammalian Gα_12/13_ subunits that interact with mammalian RGS-RhoGEFs [2], [4].

### A crystal structure of EhGα1

**Figure 4:**
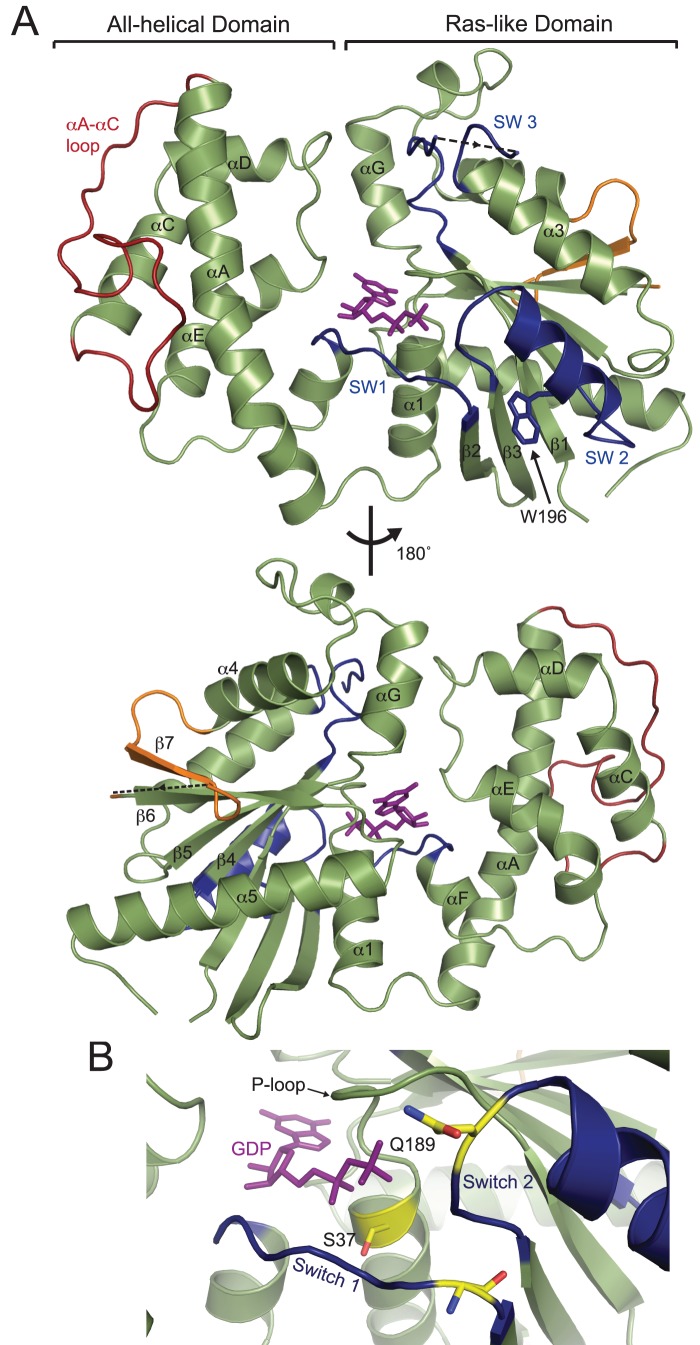
Structure of EhGα1 reveals a conserved fold with unique features. The crystal structure of EhGα1 was determined by single anomalous dispersion (SAD) using 2.6 Å resolution data (Table S1). (**A**) The EhGα1 Cα backbone is shown in green, bound to GDP in purple sticks. Conserved switch regions (SW 1–3) are dark blue. Trp-196 is solvent-exposed in the inactive state and buried when switch 2 adopts its activated conformation (e.g., Fig. 2C). Unique among Gα subunits, EhGα1 lacks an αB helix in the all-helical domain (red; labeled ‘αA-αC loop’) but possesses a unique short β-strand insert (β7) and a loop (orange) between the conserved α4 helix and β6 strand. Disordered regions in switch 3 (residues 222 and 223) and the β7- β6 loop (residues 302–310) are indicated by dashed lines. (**B**) Ser-37, conserved among Gα subunits, is an important ligand for Mg^2+^, a cofactor for GTP binding and hydrolysis. Mutation of Ser-37 to Cys is predicted to produce a dominant negative EhGα1 [40]. Gln-189 is required for orienting the nucleophilic water during GTP hydrolysis; its mutation to Leu is predicted to cripple GTPase activity, yielding a constitutively active EhGα1.

To gain better insight into the distant homology of EhGα1 versus other Gα subunits, we determined a crystal structure of EhGα1 bound to GDP by single-wavelength anomalous dispersion (SAD) using data to 2.6 Å resolution (Table S1; Fig. S6). To obtain high-quality diffracting crystals, we modified EhGα1 by removing its extended N-terminal helix (a.a. 1–22) and subjecting it to reductive lysine methylation. Neither alteration perturbed the nucleotide cycle or activation kinetics of EhGα1 (Fig. 2B,C). EhGα1 features the highly conserved Ras-like and all-helical domain structure and nucleotide-binding pocket characteristic of Gα subunits (Fig. 4A). The three switch regions are ordered in one of the two monomers in the asymmetric unit, likely due to crystal contacts (Fig. S6B). EhGα1 exhibits a highly conserved mode of nucleotide interaction, including the dispositions of residues Ser-37 and Gln-189 (Fig. 4B). The guanine ring is embraced by the conserved NKxD motif (residues 254–257; Fig. S7), with the hydrophobic portion of Lys-255 packing against the planar guanine ring. The phosphate-binding loop (P-loop) forms numerous polar contacts with the α- and β-phosphoryl groups of GDP [39].

Unique to EhGα1 is the absence of an αB helix in the all-helical domain (Fig. 4A). Although the segment between αA and αC (αA-αC loop) could be affected by crystal packing, five prolines scattered throughout this loop (positions 84, 89, 99, 103, and 106; Fig. S1A) suggest this region likely also lacks helical structure in solution. GoLoco motif-containing proteins are one of the few molecules that interact with the αB helix (*e.g.*, ref. [37]); not surprisingly, given the lack of a structurally-conserved binding site on EhGα1, the *E. histolytica* genome does not seem to encode any GoLoco motifs. EhGα1 also harbors a unique 16-residue insert in the Ras-like domain following the α4 helix (Figs. 4A, S1A). A portion of this insert forms a short β-strand (here termed β7) that extends the six-stranded β-sheet common to all heterotrimeric and Ras-family GTPases [44], [45], followed by a 15-residue loop that is disordered in our crystal structure. This region of Gα is critical for interaction with GPCRs as seen, *e.g.*, in the crystal structure of the β2 adrenergic receptor/Gs complex [46]. Because this region is important for receptor coupling and/or specificity, the existence of this insert in EhGα1 suggests a potentially unique GPCR-coupling mechanism in *E. histolytica*, but no receptor has yet been identified (see Discussion).

### G-protein signaling perturbation modulates trophozoite migration, Matrigel transmigration, and host cell attachment and killing

**Figure 5:**
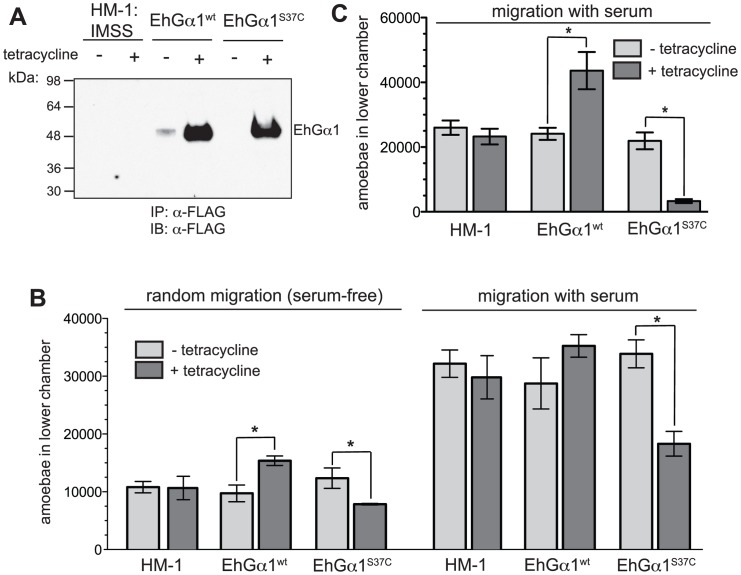
Heterotrimeric G-protein signaling increases trophozoite migration across porous membranes and Matrigel layers. (**A**) Trophozoites were stably transfected to express wildtype EhGα1 or dominant negative EhGα1^S37C^ under tetracycline control. (**B**) EhGα1^wt^-expressing trophozoites showed greater migration across a porous membrane in the absence of stimuli (serum-free) while amoebae expressing EhGα1^S37C^ showed lower migration toward both serum-free and serum-containing nutritive media. Migration of HM-1:IMSS trophozoites was not significantly different from the non-induced EhGα1^wt^ and EhGα1^S37C^ strains. Tetracycline treatment was 5 µg/mL over 24 hours. (**C**) Trophozoites expressing EhGα1^wt^ were better able to migrate through a Matrigel layer than uninduced controls. Conversely, EhGα1^S37C^ expression greatly reduced Matrigel transmigration. Parent strain HM-1:IMSS trophozoites were unaffected by tetracycline treatment and were indistinguishable from non-induced EhGα1^wt^ and EhGα1^S37C^. Error bars represent standard error of the mean. * represents statistical significance by an unpaired, two-tailed Student's t-test (p<0.05) for four independent experiments.

To determine roles of heterotrimeric G-protein signaling in pathogenesis-related behaviors of *E. histolytica*, HM-1:IMSS trophozoites were stably transfected with tetracycline-inducible expression plasmids [47] encoding either wildtype EhGα1 or the dominant negative EhGα1^S37C^ (Fig. 5A). A strain expressing the constitutively active EhGα1^Q189L^ could not be established, potentially due to cellular toxicity; however, overexpression of wildtype EhGα1 is expected to result in a moderately higher basal level of signaling to downstream components. Overexpression of signaling components is subject to limitations, including the possibility that supra-physiological expression levels and/or protein mislocalization result in toxicity or other cellular effects not typically mediated by endogenous signaling. However, this approach is useful to suggest cellular processes that may be regulated by heterotrimeric G-protein signaling and to mimic the gross perturbation that may be achieved with pharmacological agents acting on this pathway. Immunofluorescence of overexpressed EhGα1 revealed a diffuse, cytoplasmic cellular distribution that did not differ significantly between the wild type and S37C mutant strains (Fig. S8A). Endogenous EhGα1 was not assessed due to a current lack of specific antibodies. To assess potential effects of Gα subunit overexpression on trophzoite growth and viability, growth curves were assessed for the parent HM-1:IMSS, EhGα1^wt^, and EhGα1^S37C^ strains in the presence and absence of tetracycline. No significant differences in growth or viability (>90% at all time points) were observed over three days, although trophozoites expressing EhGα1^S37C^ displayed a trend toward slower growth at day 3 (Fig. S8B). All subsequent cellular experiments were conducted following growth with or without tetracycline for 24 hours.

Trophozoite motility is related to the pathogenesis of amoebic colitis, likely contributing to tissue invasion [48], [49]. Tetracycline-induced EhGα1^wt^ overexpression increased migration in the absence of a serum stimulus while EhGα1^S37C^ expression reduced migration in the presence or absence of serum in Transwell migration assays (Fig. 5B), suggesting that perturbation of heterotrimeric G-protein signaling may regulate motility at baseline and potentially in response to serum factor stimuli. However, the reduced migration of the EhGα1^S37C^ strain in the presence of serum may be due to the lower baseline trophozoite motility, as observed in the absence of a serum stimulus, rather than due to specific heterotrimeric G-protein involvement in a signaling response to serum factors. Tetracycline treatment had no measurable effect on the migration of the HM-1:IMSS parent strain or trophozoites transfected with an empty expression vector (Fig. S9A).

*E. histolytica* invades the intestinal mucosa, giving rise to ulcers and, in rare cases, systemic amoebiasis [50], [51]. To assess migration across a barrier, transfected trophozoite strains were profiled by a Transwell assay, with upper and lower chambers separated by Matrigel. Induced expression of EhGα1^wt^ enhanced, but EhGα1^S37C^ reduced, Matrigel transmigration relative to uninduced controls (Fig. 5C), revealing a potential regulatory role for heterotrimeric G-protein signaling. Tetracycline treatment had no effect on the transmigration of HM-1:IMSS or empty vector-transfected trophozoites (Fig. S9B). The effects of EhGα1^wt^ and EhGα1^S37C^ overexpression on Matrigel transmigration displayed the same trends seen for migration in the absence of serum (Fig. 5B). Thus, differential baseline migration rates may account for part or all of the observed differences in Matrigel transmigration.

**Figure 6:**
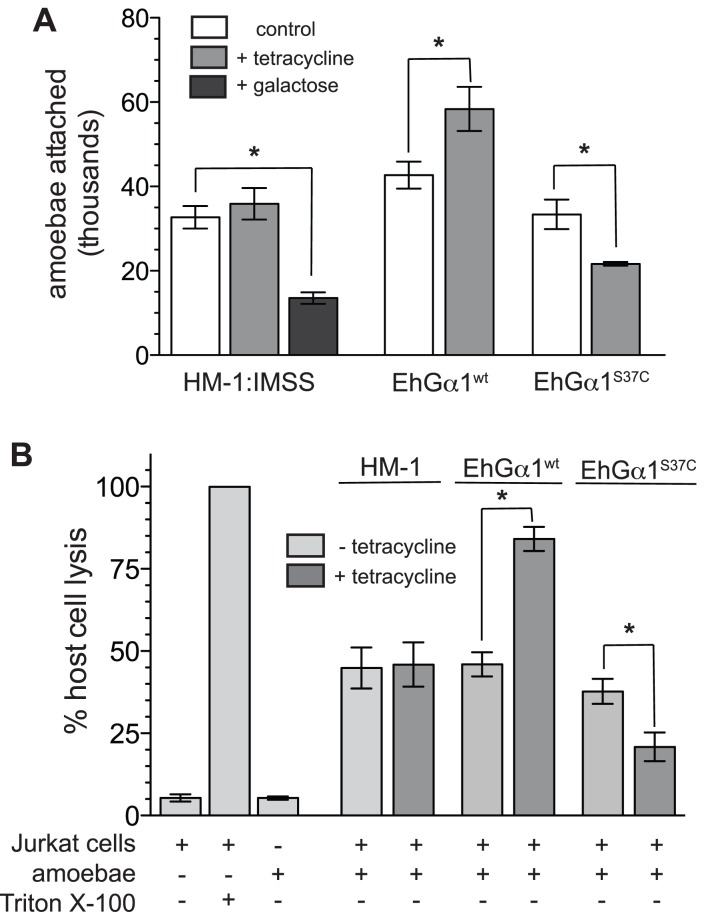
Heterotrimeric G-protein signaling positively regulates *E. histolytica* attachment to host cells as well as host cell killing. (**A**) Trophozoites attach to CHO cell monolayers, primarily through a galactose-inhibitable lectin. Overexpression of EhGα1^wt^ enhanced monolayer attachment, while expression of EhGα1^S37C^ reduced attachment. Parent strain HM-1:IMSS trophozoites were unaffected by tetracycline treatment and were indistinguishable from non-induced EhGα1^wt^ and EhGα1^S37C^. Attached trophozoites quantities were obtained by multiplying detached cell concentrations by a dilution factor. * indicates a statistically significant difference (p<0.05) between quadruplicate experiments. Error bars represent standard error of the mean. * indicates statistical significance by an unpaired, two-tailed Student's t-test (p<0.05) for four independent experiments. (**B**) Amoebae overexpressing EhGα1^wt^ or EhGα1^S37C^ displayed enhanced or reduced abilities to kill Jurkat (human T-lymphocyte) cells, respectively, as measured by LDH release in a membrane integrity assay. Cell killing by HM-1:IMSS trophozoites was not altered by tetracycline treatment. 0.5% Triton X-100 was added to Jurkat cells to define 100% host cell lysis. Tetracycline treatment was 5 µg/mL over 24 hours. Error bars represent standard error of the mean. * indicates statistical significance by an unpaired, two-tailed Student's t-test (p<0.05) for three independent experiments, with four technical replicates each.

*E. histolytica* trophozoites also attach to and kill host cells, including intestinal epithelium and responding immune cells. Host cell attachment, achieved primarily through a galactose-inhibitable lectin [13], [52], is required for subsequent cell killing. Trophozoites expressing EhGα1^wt^ displayed greater attachment to CHO cell monolayers than uninduced controls, and the opposite effect was seen in the EhGα1^S37C^ strain (Fig. 6A, S10). EhGα1^wt^ overexpression enhanced Jurkat cell killing, as assessed with a membrane integrity assay, while trophozoites expressing the dominant negative EhGα1^S37C^ were less cytotoxic (Fig. 6B). Tetracycline treatment had no effect on host cell attachment or killing by HM-1:IMSS or empty vector-transfected trophozoites (Fig. S9C, D). Thus, perturbation of heterotrimeric G-protein signaling also regulates host cell killing by *E. histolytica*. Similar patterns were observed in host cell attachment and cell killing assays; different degrees of attachment upon expression of EhGα1^wt^ or EhGα1^S37C^ may be partially or wholly responsible for the observed changes in contact-dependent cell killing.

### Regulation of transcription by perturbed heterotrimeric G-protein signaling

**Figure 7:**
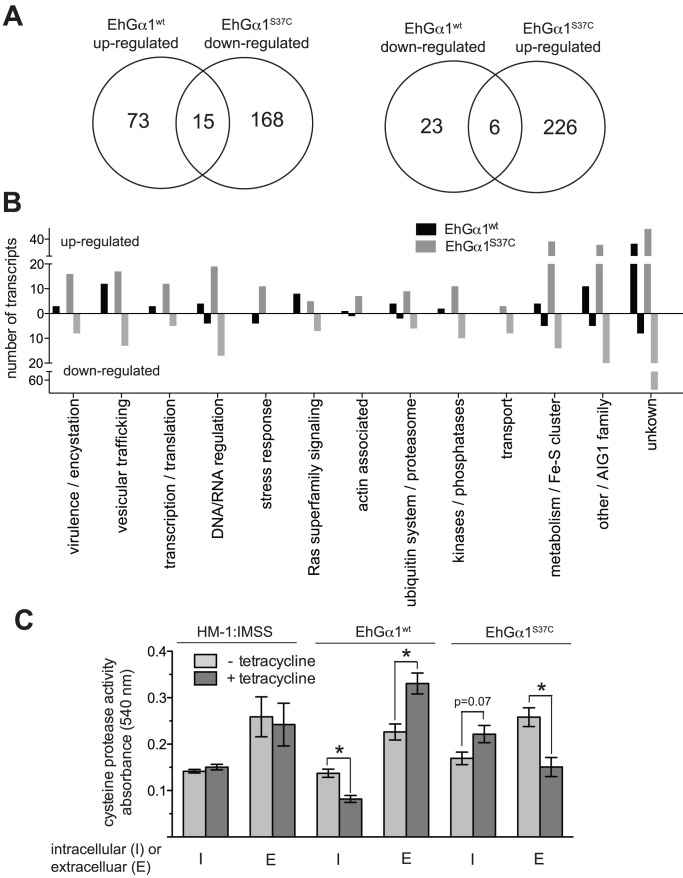
Heterotrimeric G-protein signaling alters *E. histolytica* transcription to modulate cysteine protease secretion. (**A**) 96 genes or 394 genes were differentially transcribed upon overexpression of EhGα1^wt^ or EhGα1^S37C^, respectively, when compared to uninduced controls as determined by RNA-seq. 21 transcripts were oppositely regulated in trophozoites expressing EhGα1^wt^ vs EhGα1^S37C^. (**B**) Differentially transcribed genes were categorized by putative function based on prior studies, homology to genes of known function, or predicted protein domains of known function. “Virulence/encystation” category includes genes known to modulate *E. histolytica* pathogenesis, such as cysteine proteases [48]. (**C**) Both intracellular and secreted cysteine protease activities were assessed with an azo-collagen assay. EhGα1^wt^ overexpression enhanced cysteine protease secretion, while EhGα1^S37C^ expression resulted in less extracellular (E), despite higher intracellular (I), cysteine protease activity, suggesting that transcriptional responses downstream of heterotrimeric G-protein signaling modulate *E. histolytica* pathogenic processes in part by regulating cysteine protease secretion. Tetracycline treatment in all experiments was 5 µg/mL over 24 hours. * = statistical significance by an unpaired, two-tailed Student's t-test (p<0.05) for four independent experiments.

To gain insight into potential mechanisms by which perturbation of EhGα1 expression controls pathogenesis-related behaviors in *E. histolytica*, RNA-seq was performed on mRNA isolated from trophozoites expressing EhGα1^wt^, EhGα1^S37C^, and uninduced controls. To emphasize highly transcribed genes and eliminate potential transcriptional effects of tetracycline treatment, transcripts with a *Fragments Per Kilobase of exon per Million fragments mapped* (FPKM) value less than 10 and transcripts that were up- or down-regulated (in the same direction) in both EhGα1^wt^ and EhGα1^S37C^ samples (24 hour tetracycline treatment at 5 µg/mL) relative to uninduced (tetracycline-free) trophozoites were excluded. Twenty-one genes were differentially transcribed in opposite directions upon expression of either EhGα1^wt^ or EhGα1^S37C^ (Fig. 7A). Transcriptional changes of multiple genes were verified over a 24 hour time course by RT-PCR (Fig. S11). For instance, EhGβ1 was found to be more highly expressed in trophozoites expressing EhGα1^S37C^. Analysis of putative functions for the differentially transcribed genes revealed a diversity of responses to altered heterotrimeric G-protein signaling (Fig. 7B). Stress response-related transcripts, such as those encoding heat shock proteins, were exclusively down-regulated upon EhGα1^wt^ expression and up-regulated in the dominant negative EhGα1^S37C^ strain; conversely, numerous metabolic enzymes were selectively up-regulated following expression of EhGα1^S37C^, suggesting that heterotrimeric G-protein signaling may be involved in sensing and responding to vital extracellular nutrients.

Genes with known effects on *E. histolytica* pathogenesis were also differentially transcribed, as measured by RNA-seq. (Table S2). For example, the host cell lytic factor amoebapore C was up-regulated upon EhGα1^wt^ expression, while the amoebapore A precursor was down-regulated by EhGα1^S37C^ (Table S2), consistent with the higher or lower cell killing efficiencies, respectively, of each strain (Fig. 6B) [14], [15], [48]. Down-regulation of amoebapore A upon expression of EhGα1^S37C^ was confirmed by RT-PCR at the transcriptional level, and by western blot at the protein level (Fig. S11; anti-amoebapore A was a gift from Dr. M. Leippe, U. of Kiel, Germany). A number of cysteine proteases, known factors in both host cell killing and Matrigel transmigration [53], were differentially transcribed following expression of EhGα1^S37C^ (Table S2). The down-regulation of one cysteine protease (EHI_006920) was confirmed by RT-PCR (Fig. S11). Ten Rab family GTPases, known to regulate vesicular trafficking and cysteine protease secretion [28], as well as other putative secretion/trafficking proteins, were also differentially transcribed. Specifically, four cysteine protease binding factors (CBPFs), recently shown to modulate cysteine protease secretion [54], were down-regulated in trophozoites expressing EhGα1^S37C^ (Table S2). These transcriptional effects suggested that altered cysteine protease activity and/or secretion may be a mechanism by which perturbation of heterotrimeric G-protein signaling modulates Matrigel transmigration and host cell killing (Figs. 5C & 6B). To test this hypothesis, intracellular and secreted cysteine protease activities were each measured in the EhGα1^wt^ and EhGα1^S37C^ strains. EhGα1^wt^ expression increased extracellular and decreased intracellular cysteine protease activity, likely reflecting more efficient vesicular trafficking and secretion (Fig. 7C). In contrast, EhGα1^S37C^ expression resulted in a trend toward more intracellular protease activity, although not statistically significant (p = 0.07), and significantly less extracellular protease activity relative to uninduced control trophozoites, correlating with reduced Matrigel transmigration and cell killing by this strain (Figs. 5C & 6B).

## Discussion

Here we demonstrate that functional heterotrimeric G-protein subunits are encoded by the pathogen *Entamoeba histolytica*, including single Gα and Gβ subunits, and two Gγ subunits. Like their mammalian counterparts, EhGα1, EhGβ1, and EhGγ1/2 form a nucleotide state-dependent heterotrimer. EhGα1 binds and hydrolyzes GTP and its switch regions undergo a conserved conformational change. When in an activated state, EhGα1 is seen to engage a putative effector protein, namely an RGS domain-containing RhoGEF (EhRGS-RhoGEF). EhRGS-RhoGEF likely represents a functional signaling link between heterotrimeric G-proteins and Rho family GTPases in *E. histolytica*. Indeed, Rho GTPases and other Dbl family RhoGEFs in *E. histolytica* have been implicated in multiple processes important for pathogenesis-related processes such as actin reorganization during chemotaxis, surface receptor capping, cell killing, phagocytosis, and tissue destruction [24], [25], [26], [27], [55].

The sequence of EhGα1 diverges from each of the mammalian Gα subunit subfamilies, including the Gα_12/13_ subfamily that couples to RGS-RhoGEFs. Thus EhGα1 likely represents an early evolutionary departure from the metazoan Gα/RGS-RhoGEF signaling axis, or possibly a signaling pathway of similar function with an independent evolutionary origin. A search of publicly available genome sequences using SMART [56] identified the RGS and DH-PH domain combinations exclusively in metazoan species, with the only exception being the amoebazoans. This lack of RGS-RhoGEF related proteins in non-metazoan species suggests an independent origin of the *E. histolytica* Gα/RGS-RhoGEF interaction; however, we cannot rule out the possibility that a Gα/RGS-RhoGEF interaction arose early in evolutionary history, such as an ancestral Unikonta supergroup member (*e.g.* [57]), and was later lost in fungal species, but retained in metazoans and amoebae. Among the species compared in this study, EhGα1 was found to be most similar in sequence to the *D. discoideum* Gα9, followed more distantly by *S. cerevisiae* GPA1 and GPA2, as well as *A. thaliana* GPA1. This set of Gα subunits is only loosely related by function, with *D. discoideum* Gα9 regulating cellular proliferation [8], while yeast GPA1 and GPA2 transduce signals in response to pheromones and nutrients, respectively [6]. A variety of downstream signaling machinery is utilized as well, with *S. cerevisiae* pheromone signaling occurring predominantly through Gβγ subunit effectors, while *S.c.* GPA2 engages an adenylyl cylase effector [6]. The current study clearly differentiates EhGα1 from these relatively similar Gα subunits on the sequence level, demonstrating interaction with an RGS-RhoGEF effector and no significant effect on cellular proliferation, but apparent roles in multiple pathogenesis-related processes of *E. histolytica*.

Perturbation of heterotrimeric G-protein signaling in *E. histolytica* trophozoites was observed to modulate migration, Matrigel transmigration, and host cell attachment and killing. Notably, trophozoite Matrigel transmigration is dependent on general migration to some degree, and host cell killing is dependent on attachment. Thus, the effects of heterotrimeric G-protein perturbation on Matrigel transmigration and host cell killing may be partially or wholly due to the alterations in migration and attachment, respectively. Induced expression of the dominant negative EhGα1^S37C^ impaired these pathogenic processes, suggesting that antagonizing G-protein signaling may reduce *E. histolytica* virulence. The complete mechanisms by which heterotrimeric G-proteins are linked to specific trophozoite behaviors remain to be elucidated. For instance, it is presently unclear which signaling cascades are utilized to effect transcriptional changes in response to perturbed EhGα1 expression. EhGα1 likely engages its RGS-RhoGEF effector, leading to activation of specific Rho GTPases, some of which are known to regulate cytoskeletal dynamics required for such processes as migration and Matrigel transmigration [24], [27], [55], [58]. EhGβγ may also engage as yet unidentified effectors, like its homologs in other species, leading to changes in pathogenic processes [1].

It is presently unclear how heterotrimeric G-protein signaling is activated in *E. histolytica*. Since nucleotide exchange is the rate-limiting step in the nucleotide cycle of EhGα1, an exchange factor, such as a GPCR, is likely required for high levels of EhGα1 activation. At this time, the only putative GPCR described is the Rab GTPase-binding protein EhGPCR-1 [59]. While it would be compelling to demonstrate receptor-mediated nucleotide exchange on EhGα1, our own bioinformatic analysis revealed that EhGPCR-1, while containing seven-transmembrane spanning regions, is more likely a conserved Wnt-binding factor required for Wnt secretion (as seen in *C. elegans*) [60]. Identification of a *bona fide* GPCR/ligand pair or other heterotrimeric G-protein activation mechanism in *E. histolytica* will provide powerful tools for further probing of the roles of heterotrimeric G-protein signaling in trophozoites.

## Materials and Methods

### Cloning of *E. histolytica* G-protein subunits

The open reading frame (ORF) of EhGα1 was amplified from *E. histolytica* genomic DNA (Dr. M. Vargas, Center of Investigation and Advanced Studies, Mexico City) by polymerase chain-reaction (PCR) using Phusion polymerase (New England BioLabs) and Invitrogen primers. Amplicons were subcloned using ligation-independent cloning [61] into a Novagen pET vector-based prokaryotic expression construct (“pET-His-LIC-C”) to form N-terminal tobacco etch virus (TEV) protease-cleavable, hexahistidine-tagged fusions. Mutations were made using QuikChange site-directed mutagenesis (Stratagene). ORFs of EhGα1, EhGβ1, EhGγ1, and EhGγ2, codon-optimized for mammalian cells, were obtained from Geneart (Regensburg, Germany); EhGα1 with an internal FLAG epitope, DYKDDDK inserted after His-83, was also obtained for co-immunoprecipitations. Sequences for EhGγ1 and EhGγ2, identified in genomic shotgun sequences were MSQQQLTRLLQEKERLMKNFERSKNLMKVSEACSDLVNFTKSKVDPFSPEFKDSNPWDKNNEGGCCALV and MSQQQLIRLLQEKERLMKNFERSKNLMKVSEACSELVNFTKNKIDPFSPEFKDTNPWDKSSNAGCCSLM, respectively.

### Protein purification, crystallization, and structure determination

See the Supplementary Methods for details.

### Fluorescence complementation and co-immunoprecipitation

Yellow fluorescent protein (YFP) bimolecular fluorescence complementation was performed as described [62] with modifications below. Codon-optimized ORFs of EhGγ isoforms were subcloned as HA-tagged fusions to the N-terminal 159 amino acids of YFP-venus (pcDNA3.1-YFP_N_; Dr. Nevin Lambert, MCG). The EhGβ1 ORF was subcloned as an HA-tagged fusion with a C-terminal fragment (residues 159–239) of YFP-venus (pcDNA3.1-YFP_C_; also obtained from Dr. Lambert, along with control YFP_N_-human Gγ_2_ and YFP_C_-human Gβ_1_ fusions). 200,000 COS-7 cells per well in 6-well dishes were transfected with 1 µg DNA using FuGENE-6 as per manufacturer's directions. Empty pcDNA3.1 DNA was used to maintain a constant amount of total DNA per well. Forty-eight hours post-transfection, epifluorescence was observed using an Olympus IX70 microscope with Hamamatsu monochrome CCD camera. Digital images were imported into MATLAB 2007a and quantified as previously described [62]. Pixels with greater than 40 units of intensity were considered to be fluorescent, and the percentage of positive pixels was quantified. All experiments were repeated three times. Co-immunoprecipitation was performed using the YFP-fusion proteins as previously described [62].

### Nucleotide binding, hydrolysis, and EhGα1 activation

Spontaneous GDP release, measured by [^35^S]GTPγS incorporation, and [γ-^32^P]GTP hydrolysis by single turnover assays were both quantified as previously described [37]. For GTPase acceleration assays, increasing concentrations of purified EhRGS-RhoGEF were added along with the hydrolysis-initiating magnesium. Real-time monitoring of EhGα1 tryptophan fluorescence (excitation 280 nm; emission 350 nm) was conducted as described for Gα_i1_ [37].

### Evolutionary analysis

The protein sequences of Gα subunits from humans, *S. cerevisiae*, *A. thaliana*, *D. melanogaster*, and *D. discoideum* were aligned and an unrooted phylogram derived using T-coffee [63]. Percent amino acid sequence similarities of EhGα1 and *S. cerevisiae* GPA1 were calculated relative to each human Gα subunit, using a multiple sequence alignment, as described previously [64]. The Gα family of *Drosophila melanogaster* served as a positive control for subfamily classification.

### Surface plasmon resonance

Optical detection of protein binding was conducted as described previously [65]. Briefly, His_6_-tagged EhRGS-RhoGEF was immobilized on an NTA chip surface and increasing concentrations of wildtype EhGα1 and mutants were flowed over at 10 µL/s in various nucleotide states.

### Trophozoite stable transfection

EhGα1 and EhGα1^S37C^ were subcloned with internal FLAG epitope tags into a tetracycline-inducible expression vector, described previously [47]. Axenic cultures were transfected by lipofection as previously described [66]. Briefly, amoebae at ∼5×10^6^/mL were suspended in medium 199 (Sigma) supplemented with 5.7 mM cysteine, 1 mM ascorbic acid, 25 mM HEPES (pH 6.9), 15 µg of DNA, and 30 µL of Superfect (Qiagen). After 3 hours at 37°C, trophozoites were transferred to TYI-S-33 medium overnight and selected for stable transfection with 10 µg/mL hygromycin over 3 weeks.

### Trophozoite migration and Matrigel transmigration

Trophozoite migration assays were performed essentially as described previously [67]. Briefly, amoebae were grown in the presence or absence of 5 µg/mL tetracycline for 24 hours, harvested in log growth phase, suspended in serum free TYI growth medium, and 50,000 cells loaded in the upper chamber of a Transwell migration chamber (Costar, 8 µm pore size). The lower chamber contained growth medium with or without 15% adult bovine serum. Transwell plates were incubated at 37°C for 2 hr under anaerobic conditions (GasPak EZ, BD Biosciences). Matrigel transmigration assays were performed in similar fashion, except that Matrigel was first diluted to 5 mg/mL in serum free TYI growth medium, layered on the Transwell porous filter, and allowed to gel for 6 hr prior to assay initiation. Incubation time was also extended to 16 hr to allow penetration. Migrated trophozoites attached to the lower chamber wall were detached on ice, fixed, and counted. Each experiment was performed in triplicate and statistical significance among four independent experiments was determined by an unpaired, two-tailed Student's t-test.

### Host cell attachment

Attachment of *E. histolytica* trophozoites to epithelial monolayers was assessed as previously described [68]. Chinese hamster ovary (CHO) cells were grown to confluency in 24-well plates, washed, and fixed in 4% paraformaldehyde for 30 minutes. Trophozoites (3×10^5^) grown in the presence or absence of 5 µg/mL tetracycline for 24 hours were added to the fixed monolayers in medium 199 supplemented with 5.7 mM cysteine, 1 mM ascorbic acid, and 25 mM HEPES (pH 6.9). After incubation at 37°C for 30 minutes, each well was washed gently two times with warm PBS to remove unattached trophozoites. Monolayer-attached trophozoites were detached on ice and quantified by counting with an inverted microscope. In similar experiments, trophozoites were labeled with carboxyfluorescein diacetate succinimidyl ester (CFDA-SE). Attached fluorescent trophozoites were counted in three microscopic fields at 10× magnification. Each experiment was performed in quadruplicate and statistical significance determined by an unpaired two-tailed Student's t-test.

### Cell killing

Killing of mammalian cells (Jurkat) was assessed using the CytoTox-ONE membrane integrity assay (Promega). In 96-well plates, 5×10^5^ Jurkat cells and/or 2.5×10^4^ trophozoites, grown with or without 5 µg/mL tetracycline for 24 hours, were incubated at 37°C in 200 µL of medium 199 (Sigma) supplemented with 5.7 mM cysteine, 0.5% BSA, and 25 mM HEPES pH 6.8. After 2.5 hr, 50 µL of medium from each well was incubated with Cytotox reagent and a colorimetric measure of extracellular lactate dehydrogenase activity was obtained after 10 min. 0.5% Triton X-100 was used to define 100% host cell death. Each experiment was performed with five replicates and statistical significance among three independent experiments was determined by an unpaired two-tailed Student's t-test.

### Whole transcriptome shotgun sequencing

Total RNA from 10^6^ trophozoites each of the tetracycline-induced (5 µg/mL tetracycline for 24 hours) EhGα1^wt^ and EhGα1^S37C^ strains, as well as a tetracycline-free control, was isolated using an RNeasy Mini Kit (Qiagen) per manufacturer's instructions. Duplicate RNA purifications and sequencing were obtained for each condition.

Quality of total RNA from each sample was estimated by automated electrophoresis (Bioanalyzer, Agilent). Libraries were constructed using TruSeq RNA library preparation kits (Illumina) according to manufacturer's recommendations; molarity was estimated by analysis of DNA concentration from fluorometer detection and DNA fragment size. Prepared libraries with equal molarity were pooled and used for multiplex sequencing reactions. Libraries were sequenced using 57 cycles in a single end Illumina flowcell v.3 on a HiSeq2000 instrument (Illumina) at the UNC High Throughput Sequencing Facility. Primary data analysis and demultiplexing was performed using a standard Illumina pipeline 1.8.2.

Resulting mRNA sequence reads were mapped to the annotated *Entamoeba histolytica* genome (AmoebaDB.org) using Bowtie v0.12.7 [69]. Between 12×10^6^ and 32×10^6^ reads were aligned for each sample. Aligned reads were further analyzed with Cufflinks v1.3.0 [70] and visualized using the Integrative Genomics Viewer (www.broadinstitute.org/igv/). Cuffdiff was used to determine differential expression by comparing relative transcript abundances between pairs of duplicate experiments: EhGα1^wt^ expression vs tetracycline-free control, EhGα1^S37C^ expression vs tetracycline-free control, and EhGα1^wt^ vs EhGα1^S37C^ expression. Genes exhibiting statistically significant differential transcription were compiled and corresponding annotations retrieved using software from Dr. Chung-Chau Hon (Institut Pasteur) [71]. Transcripts that were either up- or down-regulated in both the induced EhGα1^wt^ and EhGα1^S37C^ strains were excluded from further analysis, because of potential transcriptional modulation due to tetracycline treatment. Functions of the associated proteins were inferred from prior *E. histolytica* studies, by similarity to mammalian protein families, or from conserved domains of known function. All encoded proteins without annotated conservation and those with domains of unknown function were classified as “unknown”.

### Cysteine protease activity

Intracellular cysteine protease activity in amoebic lysates was assayed essentially as described previously [72]. Crude extracts of 10^6^ trophozoites, grown with or without 5 µg/mL tetracycline for 24 hours, were obtained by lysing with 5 cycles of freeze-thaw. Total protein concentration was quantified by Bradford's method. 2 mg of azo dye-impregnated collagen (Sigma) with 100 µg of crude extract in 500 µL of protease activation buffer (100 mM Tris pH 7.0 and 10 mM CaCl_2_) were incubated at 37°C for 18 hr, then terminated with 500 µL of 10% TCA. Samples were centrifuged to exclude intact collagen fibers, and supernatants collected for absorbance reading at 540 nm. In parallel experiments, the inhibitor p-hydroxy-mercuribenzoic acid (PHMB) was included at 1 mM to assess the fraction of specific cysteine protease activity. Residual protease activity (after PHMB treatment) was subtracted to determine total cysteine protease activity.

Extracellular cysteine protease activity was also assayed with azo-collagen as described above. However, 10^6^ trophozoites were incubated at 37°C for 3 hr in 500 µL PBS supplemented with 20 mM cysteine, 0.15 mM CaCl_2_, and 0.5 mM MgCl_2_, conditions known to sustain *E. histolytica* growth and allow cysteine protease secretion [53]. Following centrifugation, the cell-free conditioned medium was assayed for cysteine protease activity as above. Statistical significance was determined by an unpaired, two-tailed Student's t-test.

### Accession numbers for proteins used in this study

EhGα1, AmoebaDB EHI_140350; EhGβ1, AmoebaDB EHI_000240; glyceraldehyde-3-phosphate dehydrogenase, AmoebaDB EHI_167320; EhRGS-RhoGEF, AmoebaDB EHI_010670. EhGγ1, identified within the NCBI genomic contig AAFB02000029.1; EhGγ2, identified within the NCBI genomic contig AAFB02000157.1; amoebapore A, AmoebaDB EHI_159480; cysteine protease, AmoebaDB EHI_006920.

## Supporting Information

Figure S1 **The genome of** ***Entamoeba histolytica*** **encodes heterotrimeric G-protein subunits.** (**A**) A multiple sequence alignment of EhGα1 with selected Gα subunits from other species (Dd = *Dictyostelium discoideum*, Sc = *Saccharomyces cerevisiae*, Hs = *Homo sapiens*). The secondary structure information above the aligned sequences reflects the crystal structure of EhGα1 (this study), with naming adapted from human transducin (PDB 1TND). Residues mutated in this study are marked with black arrowheads, and gray bars indicate relative sequence identity. A 110-residue insert within Sc GPA1 (gray box) was omitted for clarity. Although a number of *E. histolytica* proteins are reportedly ADP-ribosylated by pertussis toxin [73], EhGα1 is not likely to be a substrate as it lacks the C-terminal cysteine ADP-ribosylation site shared among conventional Gα_i/o_ subunits (*e.g.*, Cys-351 in human Gα_o_). Based on the sequence of the amino terminus of EhGα1, it is likely that this protein is myristoylated on its second residue (glycine) and palmitoylated on its third residue (cysteine) [33]. (**B**) EhGβ1 is aligned with selected Gβ subunits in a fashion identical to panel A with secondary structure elements as found in transducin Gβγ (PDB 1TBG).
(EPS)

Figure S2 **Heterotrimeric G-protein signaling components are expressed in** ***E. histolytica*** **.** qRT-PCR amplification of RNA isolated from HM1 *E. histolytica* trophozoites (a kind gift of Dr. William Petri, Jr.) confirmed transcription of *EhGα1, EhGβ1, EhGγ1*, and *EhRGS-RhoGEF* genes. The basally expressed housekeeping gene *GAPDH* was included as a control. ΔC_t_ reflects the difference in threshold cycle relative to reactions lacking reverse transcriptase, used as a control for DNA contamination. Error bars represent standard error of the mean.
(EPS)

Figure S3 **Example bimolecular fluorescence complementation micrographs.** YFP fluorescence was detected microscopically in COS-7 cells expressing heterotrimeric G-protein subunits. YFP complementation was observed when EhGα1 was co-expressed with EhGβ1 and EhGγ1 (**A, B**) or EhGγ2 (**C, D**). The human subunits Gβ1 and Gγ_2_ exhibited complementation, while the expressed N- and C-terminal fragments of YFP did not (**E, F**). For a quantification of fluorescence, see Figure 1.
(EPS)

Figure S4 **The inactive EhGα1(S37C) constitutively binds to EhGβ1γ2, while the constitutively active EhGα1(Q189L) mutant does not.** Co-immunoprecipitations of EhGα1 and mutants with EhGβ1 and EhGγ2 were conducted as in Figure 1. As predicted, the dominant negative S37C mutant remains bound to EhGβ1γ2, even in excess GTPγS. The constitutively active, GTPase-deficient Q189L mutant does not bind EhGβ1γ2 in either nucleotide state.
(EPS)

Figure S5 **Mammalian Gα subfamily homology analyses.** Sequence similarity to human Gα subunits was plotted for the Gα subunits from *Drosophila melanogaster* (**A**), *Saccharomyces cerevisiae* GPA1, and EhGα1 (**B**). In contrast with *D. melanogaster* subunits, EhGα1 cannot be classified as a member of any particular Gα subfamily.
(EPS)

Figure S6 **Structural comparison of EhGα1 with** ***Hs*** **transducin and switch 2 crystal contacts.** (**A**) The two EhGα1 molecules in the asymmetric unit are highly similar, although switch 2 of chain B (wheat) is partially disordered. (**B**) Crystal contacts between the ordered switch 2 of chain A (blue) and a neighboring molecule (orange) likely account for the structural differences between the two molecules in the asymmetric unit. The non-polar Trp-196 and N-dimethyl lysine-195 (MLY-195) interface with a hydrophobic patch on a neighboring molecule. Switch 2 may be drawn away from the nucleotide pocket, accounting for the absence of bound AlF_4_^−^ (see discussion below). (**C, D**) The model of EhGα1 is superposed with human transducin in two nucleotide states (slate blue, AMF, PDB 1TAD; teal, GDP, PDB 1TAG). EhGα1 lacks an αB helix seen in transducin and all other Gα subunits and contains a unique α4-β6 insert (orange). Switch 2 of EhGα1 (chain A) adopts a distinct conformation from both the active and inactive forms of transducin, likely due to crystal contacts with a neighboring molecule.
(EPS)

Figure S7 **Electron density map of guanine nucleotide binding pocket of EhGα1.** A region of the 2F_o_-F_c_ electron density map is shown in stereo view from the structure of EhGα1 (*yellow sticks*) bound to GDP (*purple sticks*). The nucleotide binding pocket is highly similar to mammalian Gα subunits, featuring a conserved phosphate binding loop (P-loop; Glu-33 shown) and an NKxD motif (residues 254–257). Switch one also directly contacts the nucleotide, and Arg-163 forms polar contacts with the P-loop Glu-33.
(EPS)

Figure S8 **Expression of EhGα1^wt^ or EhGα1^S37C^ does not significantly alter trophozoite proliferation.** (**A**) The cellular distribution of overexpressed FLAG-EhGα1^wt^ and FLAG-EhGα1^S37C^ were assessed by immunofluorescence with a Cy3 anti-FLAG conjugate. Both wild type and mutant EhGα1 exhibited similar diffusely cytoplasmic localizations following induced expression by treatment with 5 µg/mL tetracycline for 24 hr. Nuclei were stained with DAPI. (**B**) Trophozoites of the parent HM-1:IMSS, EhGα1^wt^, and EhGα1^S37C^ strains were seeded in TYI medium with or without 5 µg/mL tetracycline and cell numbers assessed over 3 days. Cell viability was >90% at each measurement, as determined by trypan blue dye exclusion. No significant differences in growth were identified among the strains, although trophozoites induced to express EhGα1^S37C^ trended toward slower growth at day 3. Error bars represent standard error of the mean for three independent experiments.
(EPS)

Figure S9 ***E. histolytica*** **transfected with empty vector is not affected by tetracycline treatment.** HM-1:IMSS trophozoites were stably transfected with empty tetracycline-inducible expression vector. (**A**) Transwell migration and (**B**) Matrigel transmigration of parent strain and vector-transfected trophozoites did not differ significantly upon tetracycline treatment of 24 hours prior to the assay. Similarly, transfection with empty vector and tetracycline treatment had no significant effect on host cell attachment (**C**) or host cell killing (**D**). Error bars represent standard error of the mean for four independent experiments in panels A–C and three independent experiments in panel D. Statistical significance was tested using an unpaired, two-tailed Student's t-test.
(EPS)

Figure S10 **Microscopic analysis of perturbed** ***E. histolytica*** **attachment to host cells upon overexpression of EhGα1^wt^ or EhGα1^S37C^.** (**A**) Trophozoites grown in the presence or absence of 5 µg/mL tetracycline were fluorescently labeled with CFDA and allowed to attach to fixed, confluent layers of CHO cells. Phase contrast (upper panels) and epifluorescence (lower panels) images were obtained of attached trophozoites. (**B**) Attachment was quantified by counting trophozoites in three microscopic fields (10×). Overexpression of EhGα1^wt^ enhanced monolayer attachment, while expression of EhGα1^S37C^ reduced attachment. Parent strain HM-1:IMSS trophozoites were unaffected by tetracycline treatment and were indistinguishable from non-induced EhGα1^wt^ and EhGα1^S37C^. Error bars represent standard error of the mean. * represents statistical significance by an unpaired, two-tailed Student's t-test (p<0.05) for three independent experiments.
(EPS)

Figure S11 **RT-PCR analysis of differentially transcribed genes and altered expression of amoebapore A protein.** (**A**) qRT-PCR amplification of RNA isolated from HM1 *E. histolytica* trophozoites confirmed differential transcription of EhGα1, EhGβ1, amoebapore A, and a cysteine protease (EHI_006920) upon tetracycline treatment of the parent HM-1:IMSS, EhGα1^wt^, or EhGα1^S37C^ strains over 24 hours. * indicates statistically significant difference from time zero (no tetracycline exposure), using an unpaired, two-tailed Student's t-test for two technical duplicates of two independent experiments. EhGα1 expression was significantly up-regulated in the EhGα1^wt^ and EhGα1^S37C^ strains, while EhGβ1 was up-regulated and amoebpore A and cysteine protease (EHI_006920) were down-regulated upon expression of EhGα1^S37C^. (**B**) Trophozoite lysates were subjected to western blotting with anti-amoebapore A (kind gift of M. Leippe, U. of Kiel, Germany), with actin serving as a loading control. Amoebapore A protein expression is reduced in parallel with its transcriptional downregulation upon overexpression of EhGα1^S37C^.
(EPS)

Table S1 **Data collection and refinement statistics for lysine-methylated selenomethionine EhGα1.**
(PDF)

Table S2 **Genes differentially transcribed in** ***E. histolytica*** **trophozoites expressing EhGα1 or EhGα1^S37C^ with known roles in pathogenesis or putative vesicular trafficking functions.**
(PDF)

Text S1 **Supplementary materials and methods.**
(DOC)
