# Supplementary material for: Heterotrimeric G-protein Signaling Is Critical to Pathogenic Processes in Entamoeba histolytica
Source: PLoS Pathog. 2012 Nov 15;8(11):e1003040. doi: 10.1371/journal.ppat.1003040 (PMC3499586; doi:10.1371/journal.ppat.1003040)
Supplement: Table S1 — Data collection and refinement statistics for lysine-methylated selenomethionine EhGα1. (PDF) [file ppat.1003040.s012.pdf]

Table S1. Data collection and refinement statistics for lysine-methylated selenomethionine EhGα1.

	EhGα1·GDP
**PDB accession code**	4FID
**Data collection**	
Space group	$P2_12_12_1$
Cell dimensions	
a, *b*, *c* (Å)	56.3, 56.9, 229.8
α, β, γ (°)	90, 90, 90
	Peak
Wavelength (Å)	0.97954
Resolution (Å)	45.9 - 2.60 (2.62 – 2.60)*
No. unique reflections	43,503
R_{merge} (%)	7.7 (98.2)**
I / σI	30.7 (2.6)
Completeness (%)	99.3 (94.0)
Redundancy	13.3 (13.3)
**Refinement**	
Resolution (Å)	45.9 – 2.6 (2.7 – 2.6)
No. reflections	43503 (4336)
R_{work} / R_{free} (%)	18.9 / 25.8 (25.8 / 36.4)
No. atoms	5013
Protein	4905
Ligand/ion	56
Water	39
B-factors (Å^2)	
Protein	58.1
Ligand/ion	57.4
Water	44.8
R.m.s deviations	
Bond lengths (Å)	0.008
Bond angles (°)	1.205

*Values in parentheses are for highest-resolution shell.
**All data were collected from a single crystal.
