# Supplementary material for: Heterotrimeric G-protein Signaling Is Critical to Pathogenic Processes in Entamoeba histolytica
Source: PLoS Pathog. 2012 Nov 15;8(11):e1003040. doi: 10.1371/journal.ppat.1003040 (PMC3499586; doi:10.1371/journal.ppat.1003040)
Supplement: Table S2 — Genes differentially transcribed in E. histolytica trophozoites expressing EhGα1 or EhGα1S37C with known roles in pathogenesis or putative vesicular trafficking functions. (PDF) [file ppat.1003040.s013.pdf]

Table S2. Genes differentially transcribed in *E. histolytica* trophozoites expressing EhGα1 or EhG$\alpha 1^{S37C}$ with known roles in pathogenesis or putative vesicular trafficking functions.

Gene name	AmoebaDB accession no.	fold change upon EhGα1 expression (p value)	fold change upon EhG$\alpha 1^{S37C}$ expression (p value)
**Virulence genes**			
amoebapore C	EHI_118270	2.6 (0.002)	
myosin II heavy chain	EHI_014010	2.4 (< 0.001)	
vacuolar ATP synthase	EHI_029370	1.7 (0.001)	
EhRGS-RhoGEF	EHI_010670	2.2 (0.001)	
amoebapore A precursor	EHI_159480		-1.6 (0.004)
cysteine protease	EHI_062480		-2.2 (< 0.001)
cysteine protease	EHI_126170		-1.5 (0.021)
cysteine protease	EHI_138460		-1.5 (0.007)
cysteine protease	EHI_006920		-2.5 (< 0.001)
cysteine protease	EHI_045290		1.5 (0.010
cysteine protease	EHI_064430		2.3 (< 0.001)
Gal/GalNac lectin light subunit	EHI_049690		1.6 (0.005)
Gal/GalNac lectin intermediate subunit	EHI_183000		2.6 (< 0.001)
EhGβ	EHI_000240		1.6 (0.004)
**Rab family GTPases**			
Rab GTPase	EHI_168450	1.9 (0.002)	-1.9 (0.001)
Rab GTPase (RabK5)	EHI_012380	1.5 (0.011)	
Rab GTPase (RabX22B)	EHI_014060	1.6 (0.004)	
hypothetical protein (Rab domain)	EHI_014210	1.8 (< 0.001)	
Rab GTPase (RabI1)	EHI_177550	2.3 (< 0.001)	
Rab GTPase (Rab7C)	EHI_189990		-161.1 (< 0.001)
Rab GTPase (Rab11A)	EHI_005460		2.3 (< 0.001)
Rab GTPase (Rab11D)	EHI_056100		2.4 (< 0.001)
Rab GTPase (Rab2C)	EHI_067850		2.0 (< 0.001)
Rab11B	EHI_107170		10.3 (< 0.001)
**Other secretion/trafficking proteins**			
VHS domain containing protein	EHI_087590	2.1 (< 0.001)	-1.7 (0.002)
hypothetical protein (DENN domain)	EHI_014090	1.8 (< 0.001)	-1.6 (< 0.001)
hypothetical protein (DENN domain)	EHI_010320	1.8 (0.006)	
Rab GTPase activating protein	EHI_060420	2.6 (< 0.001)	
ankyrin	EHI_004990	2.1 (< 0.001)	
hypothetical protein (SFT2-like)	EHI_200980	1.9 (0.006)	
MIT domain protein	EHI_093860	1.8 (0.007)	
Rab GTPase activating protein	EHI_170310		-1.5 (0.018)
TBC1 domain family RabGAP 5	EHI_058320		-2.0 (< 0.001)
ankyrin repeat protein	EHI_134800		-1.7 (0.019)
hypothetical protein (M6PR binding)	EHI_161980		-2.1 (< 0.001)
kinesin motor protein	EHI_196390		-1.8 (< 0.001)
cysteine protease binding factor 2	EHI_087660		-2.0 (< 0.001)
cysteine protease binding factor 3	EHI_161650		-1.7 (0.001)
cysteine protease binding factor 4	EHI_012340		-1.6 (0.005)
cysteine protease binding factor 5	EHI_137940		-2.3 (< 0.001)
hypothetical protein (DENN domain)	EHI_135120		2.9 (0.002)
hypothetical protein (TBC/RabGAP)	EHI_189120		2.0 (0.005)
vesicle-fusing ATPase	EHI_004640		2.4 (< 0.001)
adaptor protein (AP) family protein	EHI_023600		1.9 (0.001)
AP-3 complex subunit delta 1	EHI_158840		2.2 (< 0.001)
AP-3 complex subunit	EHI_164810		2.1 (< 0.001)
dynamin-like protein	EHI_052740		2.6 (< 0.001)
clathrin-adaptor medium chain	EHI_099240		2.5 (< 0.001)
coatamer beta subunit	EHI_088220		2.2 (0.002)
coatamer beta subunit	EHI_173180		2.4 (0.018)
coatamer beta subunit	EHI_173390		2.6 (< 0.001)
Sec1 family protein	EHI_093130		2.2 (< 0.001)
hypothetical protein (VHS domain)	EHI_152920		1.8 (0.003)
