# Supplementary material for: Heterotrimeric G-protein Signaling Is Critical to Pathogenic Processes in Entamoeba histolytica
Source: PLoS Pathog. 2012 Nov 15;8(11):e1003040. doi: 10.1371/journal.ppat.1003040 (PMC3499586; doi:10.1371/journal.ppat.1003040)
Supplement: Text S1 — Supplementary materials and methods. (DOC) [file ppat.1003040.s014.doc]

**Supplementary Methods and Materials**

**Protein purification**

Human Gα_i1_ was purified as described [3]. For hexahistidine-tagged EhGα1 and EhRGS-RhoGEF, B834 E. coli were grown to an OD_600nm_ of 0.7−0.8 at 37°C before induction with 700 μM or 1 mM isopropyl-β-D-thiogalactopyranoside (IPTG), respectively, for 14-16 hours at 20°C. Cell pellets were resuspended in N1 buffer (for EhGα1: 50 mM Tris-HCl, pH 8.0, 300 mM NaCl, 10 mM MgCl_2_, 10 mM NaF, 30 μM AlCl_3_, 50 μM GDP, 30 mM imidazole, 5% (w/v) glycerol; for EhRGS-RhoGEF: 50 mM HEPES pH 8.0, 150 mM NaCl, 30 mM imidazole, 5% (w/v) glycerol) and lysed at 10,000 kPa using an Avestin Emulsiflex. Cleared lysates were applied to nickel-nitrilotriacetic acid resin (GE Healthcare), washed, and eluted with N1 buffer containing 300 mM imidazole. Eluted protein was resolved using a calibrated size exclusion column (GE Healthcare) with S200 buffer for EhGα1 (50 mM Tris-HCl pH 8.0, 250 mM NaCl, 5 mM DTT, 5% (w/v) glycerol, and 50 mM GDP) or EhRGS-RhoGEF (50 mM Tris-HCl pH 8.0, 250 mM NaCl, 5 mM DTT, 5% (w/v) glycerol). Comparison of global fold between wild type and E39K mutant EhRGS-RhoGEF was performed using circular dichroism as previously described [4].

For crystallization, the flexible N-terminal helix (22 residues) was removed from EhGα1; similar alterations in human Gα aided crystallization while not perturbing the resulting structure [3]. For anomalous dispersion, a selenomethionine derivative was produced using selenomethionine-containing minimal media (Molecular Dimensions). Selenomethionine EhGα1ΔN was purified by nickel-NTA chromatography as above, TEV protease cleavage and anion exchange chromatography, as described for Gα_i1_ [3]. Prior to gel filtration, EhGα1ΔN was subjected to reductive alkylation with a dimethylamine-borane complex, exactly as described [5]. Selenomethionine EhRGS-RhoGEF lacking the first two and last two residues (a.a. 3-517) was purified by nickel-NTA chromatography as above, with TEV protease cleavage of the affinity tag and a second nickel-NTA chromatography step prior to gel filtration.

**Crystallization and structure determination**

Crystals of lysine-methylated selenomethionine EhGα1ΔN (lacking 22 N-terminal residues) were obtained by vapor diffusion from hanging drops at 18°C. EhGα1ΔN at 15 mg/mL in crystallization buffer (50 mM HEPES pH 6.5, 150 mM NaCl, 10 mM MgCl_2_, 10 mM NaF, 30 μM AlCl_3_, and 50 μM GDP) was mixed 1:1 and equilibrated against crystallization solution containing 1.5 M ammonium sulfate, 175 mM K/Na tartrate, and 100 mM sodium citrate pH 5.6. Hexagonal rod crystals grew to 300 x 40 x 20 μm over 21 days. EhGα1ΔN crystals displayed the symmetry of space group P2_1_2_1_2_1_ (a = 56.3 Å, b = 56.9 Å, c = 229.8 Å, α = β = γ = 90°), with two monomers in the asymmetric unit. Prior to data collection, crystals were serially transferred for ~30 seconds to well solution supplemented with 25% (v/v) glycerol at 5% increments and plunged into liquid nitrogen.

Anomalous diffraction data were obtained at 0.97954 Å wavelength (selenium absorption peak) and 100K temperature at the GM/CA-CAT ID-D beamline (APS, Argonne National Labs) and processed using HKL2000 [6]. Since the EhGα1 crystals were sensitive to radiation, a highly redundant dataset was assembled by combining partial datasets collected at seven points along a single rod-shaped crystal. Heavy atom searching, experimental phasing, and automated model building used Phenix AutoSol [7]. Heavy atom searching identified 26 of 26 possible sites, and refinement yielded an estimated Bayes correlation coefficient of 59.7 to 2.8 Å resolution. After density modification, the estimated Bayes correlation coefficient increased to 59.8. ~80% of the model was constructed automatically, and the remaining portion was built manually. The current model (Supplementary Table S1) contains two EhGα1 monomers bound to GDP; AlF_4_^-^ could not be located in the electron density map and thus does not seem to be incorporated, despite the presence of AlCl_3_, MgCl_2_, and NaF (AMF) in the crystallization buffer.

Refinement was carried out against peak anomalous data with Bijvoet pairs kept separate using phenix.refine [7] interspersed with manual model revisions using the program Coot [8] and consisted of conjugate-gradient minimization and calculation of individual atomic displacement and translation/libration/screw (TLS) parameters [9]. Residues that could not be identified in the electron density were: chain A, 19-21, 221-222, 304-317, 356-358; chain B, 19-22, 188-196, 219-224, 302-313, 354-358. The model exhibits excellent geometry as determined by MolProbity [10]. A Ramachandran analysis identified 95.2% favored, 4.8% allowed, and 0% disallowed residues. A refined molecular replacement solution of diffraction data to 3.3 Å from native, methionine-containing EhGα1 crystals showed no noticeable differences at this resolution. The selenomethionine EhGα1 data, of higher quality and resolution, were used for refinement of the current model. Coordinates and structure factors are deposited in the RCSB Protein Data Bank (id 3RKA).

Crystals of EhGα1 were obtained only in the presence of GDP·AMF, but no electron density was observed for the AlF_4_^-^, and thus and thus our structure is representative of the inactive, GDP-bound state. Part of switch 3 in both monomers of the asymmetric unit and most of switch 2 in chain B are disordered, which is characteristic of the Gα inactive state in the absence of Gβγ binding [11,12]. Switch 2 forms a well-ordered helix in chain A (Fig. 4) with Trp-196 in a solvent-exposed position, also indicative of the inactive state. This conserved tryptophan rotates into a hydrophobic pocket upon activation by either GTP or AMF (Fig. 2, S6; [3]). Interestingly, the switch 2 region of EhGα1 occupies a position intermediate between that seen for human transducin in either the GDP- or AMF-bound forms (Fig. S6C,D; PDB 1TAG and 1TAD). The unique conformation of switch 2 in EhGα1 is likely a result of crystal contacts; Trp-196 and the N-dimethylated Lys-195 interact with a hydrophobic patch on the neighboring molecule (Fig. S6B). These crystal contacts may pull switch 2 away from the nucleotide pocket, potentially allowing AlF_4_^-^ to dissociate from EhGα1 and thus accounting for its absence in the electron density map. Another example of a GDP-only bound Gα structure despite crystallization in the presence of GDP·AMF is human Gα_13_ [12]. The authors hypothesize that low-pH crystallization conditions are unfavorable for AlF_4_^-^ binding, which is corroborated by the study presented here as our EhGα1 crystals were grown at low pH (~4.5) as well.

**qRT-PCR of *E histolytica* gene transcription**

RNA from ~1 x 10^6^ *E. histolytica* HM1:IMSS trophozoites (Dr. William Petri Jr., UVa) was isolated by TRIzol Reagent (Invitrogen) and phenol extraction as per manufacturer’s instruction. RT-PCR was conducted exactly as in [13], with and without added reverse transcriptase (RT) to control for genomic DNA contamination, since the target *E. histolytica* transcripts lacked suitable intron/exon boundaries. The number of cycles until threshold (C_t_) was determined using an ABI Prism 7700. The threshold cycle number of each gene in RT-negative control reactions was subtracted from the C_t_ value for each transcript in RT-containing reactions, yielding ΔC_t_.

For timecourse validation of RNA-seq results, RNA was isolated (RNeasy, Qiagen) from uninduced trophozoites (time zero) or trophozoites exposed to 5 μg/mL of tetracycline for 6, 12, or 24 hours. RT-PCR was conducted and threshold cycles determined exactly as for detection of signaling component transcripts, above. Changes in transcript levels were determined by the 2^-ΔΔCt^ method as described previously [13], with the time zero average being assigned a 100% value. Each time point represents duplicate RT-PCR reactions conducted on duplicate biological samples, and statistically significant difference from time zero determined by an unpaired, two-tailed Student’s t-test.

qRT-PCR primers (5’->3’ direction) for quantitation of *E. histolytica* gene expression were as follows: GAPDH, forward CATATTAAGGGAGGAGCTAAGA, reverse ATGCCTCAGTGTTAACTCCA, probe F-TCAGCCCCATCTGCTGATGCACCA-Q; EhGβ1, forward TTCGTATTAGAAGTTCATGGGT, reverse GGACACAATATTATCAAGACCAC, probe F-TGACATGTGCCTATGCCCCTTCTATG-Q; EhGα1, forward CCTAAAAGTAAAGAATTTACTACAG, reverse CATCTGCCCAAAGTGCTTCA, probe F-ACCCTGTTACTCTTCCATTTTCACCAG-Q; EhGγ1, forward AACTTCGAGAGATCAAAGAACT, reverse CAGGACTGAAAGGGTCTACT, probe F-TGAAAGTAAGTGGAGCATGTTCTGAATTAG-Q; amoebapore A, forward GGAGCAGTTGATAAAGTAACTGA, reverse CCATATGAAACAATCTTTGTGCA, probe F-CACTCTGTGCTAAAGCAGATGGTCTTG-Q; cysteine protease (EHI_006920), forward AGAAGCACTAACTCCAGTAAAG, reverse CTTTCAAGAAATCCAATAGCAGC, probe F-AGCCCAACATGTCCCTCTTGAAAATTG-Q. “F” represents fluorescein, or 5’-tetrachloro-fluorescein (TET) in 18S, and “Q” represents the quencher, TAMRA.

**Western blotting**

Trophzoites in the logarithmic growth phase were harvested and washed three times in PBS. For assessment of amoebapore A expression, trophozoites were grown in the presence or absence of 5 μg/mL tetracycline for 24 hours prior to harvesting. Trophozoites were lysed in buffer containing 20 mM Tris pH 7.5, 100 mM NaCl, 5 mM MgCl_2_, 1 mM EDTA, 1% (v/v) NP-40 alternative (Calbiochem), 0.25% deoxycholate, and protease inhibitors (Complete Mini, Roche), and insoluble fractions were removed by centrifugation. For assessment of FLAG-EhGα1 overexpression, soluble lysates were incubated overnight with anti-FLAG affinity gel (Sigma) prior to elution in SDS buffer and protein separation by SDS-PAGE. Proteins were transferred to nitrocellulose membranes and immunoblotted with an anti-FLAG HRP conjugate (M2 monoclonal antibody, Sigma), a polyclonal anti-amoebapore A (kind gift of Dr. M. Leippe, Kiel, Germany), or an HRP-conjugated anti-β actin (cat. # sc-47778, Santa Cruz).

**Immunofluorescence microscopy**

Trophozoites were harvested in the log growth phase following 24 hours incubation in the presence or absence of 5 μg/mL tetracycline. Amoebae were allowed to adhere to poly-D-lysine coated coverslips, fixed with 4% paraformaldehyde in phosphate buffered saline (PBS), permeabilized with 0.3% Triton X-100 in PBS, and blocked with 1% bovine serum albumin (BSA). The trophozoites were then immunostained with Cy3-conjugated monoclonal anti-FLAG antibody (Sigma), nuclei stained with DAPI, and images obtained with an Olympus IX70 microscope with Hamamatsu monochrome CCD camera.

**Trophozoite growth curves**

Trophozoites were grown in the log phase in the presence or absence of 5 μg/mL tetracycline for 24 hours prior to the harvesting and seeding of 5 x 10^5^ amoebae into a 25 cm^2^ flask. Tetracycline-induced strains were maintained in growth medium supplemented with 5 μg/mL tetracycline throughout the experiment. Cells were removed and counted on a hemocytometer at 12, 24, 48, and 72 hours to assess proliferation. At each time point, trophozoite viability was measured by trypan blue exclusion. Each experiment was independently performed three times and statistical significance determined by an unpaired, two-tailed Student’s t-test.

**References**

1. Soid-Raggi LG, Torres-Marquez ME, Meza I (1998) Entamoeba histolytica: identification of functional Gs and Gi proteins as possible signal transduction elements in the interaction of trophozoites with fibronectin. Exp Parasitol 90: 262-269.

2. Wedegaertner PB, Wilson PT, Bourne HR (1995) Lipid modifications of trimeric G proteins. J Biol Chem 270: 503-506.

3. Bosch DE, Kimple AJ, Sammond DW, Muller RE, Miley MJ, et al. (2010) Structural determinants of affinity enhancement between GoLoco motifs and G-protein alpha subunit mutants. J Biol Chem 286: 3351-3358.

4. Johnston CA, Afshar K, Snyder JT, Tall GG, Gonczy P, et al. (2008) Structural determinants underlying the temperature-sensitive nature of a Galpha mutant in asymmetric cell division of Caenorhabditis elegans. J Biol Chem 283: 21550-21558.

5. Rayment I (1997) Reductive alkylation of lysine residues to alter crystallization properties of proteins. Methods Enzymol 276: 171-179.

6. Otwinowski ZaWM (1997) Processing of X-ray Diffraction Data Collected in Oscillation Mode. Methods in Enzymology. New York: Academic Press. pp. 307-326.

7. Adams PD, Afonine PV, Bunkoczi G, Chen VB, Davis IW, et al. (2010) PHENIX: a comprehensive Python-based system for macromolecular structure solution. Acta Crystallogr D Biol Crystallogr 66: 213-221.

8. Emsley P, Cowtan K (2004) Coot: model-building tools for molecular graphics. Acta Crystallogr D Biol Crystallogr 60: 2126-2132.

9. Painter J, Merritt EA (2006) Optimal description of a protein structure in terms of multiple groups undergoing TLS motion. Acta Crystallogr D Biol Crystallogr 62: 439-450.

10. Chen VB, Arendall WB, 3rd, Headd JJ, Keedy DA, Immormino RM, et al. (2010) MolProbity: all-atom structure validation for macromolecular crystallography. Acta Crystallogr D Biol Crystallogr 66: 12-21.

11. Mixon MB, Lee E, Coleman DE, Berghuis AM, Gilman AG, et al. (1995) Tertiary and quaternary structural changes in Gi alpha 1 induced by GTP hydrolysis. Science 270: 954-960.

12. Kreutz B, Yau DM, Nance MR, Tanabe S, Tesmer JJ, et al. (2006) A new approach to producing functional G alpha subunits yields the activated and deactivated structures of G alpha(12/13) proteins. Biochemistry 45: 167-174.

13. Kim HS, Lee G, John SW, Maeda N, Smithies O (2002) Molecular phenotyping for analyzing subtle genetic effects in mice: application to an angiotensinogen gene titration. Proc Natl Acad Sci U S A 99: 4602-4607.
